# Persistence of Only a Minute Viable Population in Chlorotic *Microcystis aeruginosa* PCC 7806 Cultures Obtained by Nutrient Limitation

**DOI:** 10.1371/journal.pone.0133075

**Published:** 2015-07-16

**Authors:** Diogo de Abreu Meireles, Jan Schripsema, Andrea Cristina Vetö Arnholdt, Denise Dagnino

**Affiliations:** 1 Laboratório de Biotecnologia, Universidade Estadual do Norte Fluminense Darcy Ribeiro, Campos dos Goytacazes, Rio de Janeiro, Brazil; 2 Grupo Metabolômica, Laboratório de Ciências Químicas, Universidade Estadual do Norte Fluminense Darcy Ribeiro, Campos dos Goytacazes, Rio de Janeiro, Brazil; 3 Laboratório de Biologia do Reconhecer, Universidade Estadual do Norte Fluminense Darcy Ribeiro, Campos dos Goytacazes, Rio de Janeiro, Brazil; University of Freiburg, GERMANY

## Abstract

Cultures from the cyanobacterial strain *Microcystis aeruginosa* PCC 7806 submitted to nutrient limitation become chlorotic. When returned to nutrient rich conditions these cultures regain their green colour. The aim of this study was to verify whether the cells in these cultures could be considered resting stages allowing the survival of periods of nutrient starvation as has been reported for *Synechococcus* PCC 7942. The experiments with *Microcystis* were carried out in parallel with *Synechococcus* cultures to rule out the possibility that any results obtained with *Microcystis* were due to our particular experimental conditions. The results of the experiments with *Synechococcus* PCC 7942 cultures were comparable to the reported in the literature. For *Microcystis* PCC 7806 a different response was observed. Analysis of chlorotic *Microcystis* cultures by flow cytometry showed that the phenotype of the cells in the population was not homogenous: the amount of nucleic acids was about the same in all cells but only around one percent of the population emitted red autofluorescence indicating the presence of chlorophyll. Monitoring of the reversion of chlorosis by flow cytometry showed that the re-greening was most likely the result of the division of the small population of red autofluorescent cells originally present in the chlorotic cultures. This assumption was confirmed by analysing the integrity of the DNA and the membrane permeability of the cells of chlorotic cultures. Most of the DNA of these cultures was degraded and only the autofluorescent population of the chlorotic cultures showed membrane integrity. Thus, contrary to what has been reported for other cyanobacterial genera, most of the cells in chlorotic *Microcystis* cultures are not resting stages but dead. It is interesting to note that the red autofluorescent cells of green and chlorotic cultures obtained in double strength ASM-1 medium differ with respect to metabolism: levels of emission of red autofluorescence are higher in the cells of green cultures and the ability to convert fluorescein diacetate of these cells are heterogeneous when compared to the autofluorescent cells of chlorotic cultures. Thus, the small population of the red autofluorescent cells of chlorotic cultures are in a differentiated metabolic state that allow them to persist in conditions in which most of the population loses viability; persistent cells can be detected in chlorotic cultures maintained for more than a year.

## Introduction

The cyanobacteria form a monophyletic group comprising the oxygenic photosynthetic bacteria [1]. They are inhabitants of many types of environments and comprise organisms capable of living under extreme conditions of temperature, radiation intensity and water availability [2], [3] and [4]. Cyanobacteria are among the few organisms able to inhabit several of the earth’s most extreme environments like the core of the Atacama Desert, arctic deserts and hot springs.

Usual inhabitants of extreme environments, under certain unfavourable circumstances for growth, specialised resting cells (akinetes) can develop from vegetative cells. Like the spores of other bacteria, when compared to vegetative cells, akinetes are more resistant to environmental stresses. Akinetes have been shown to be resistant to drying, freezing and long-term storage in anoxic sediments [1].

Adaptations of vegetative cells that allow cyanobacteria to survive inadequate growth conditions have also been reported and include desiccation tolerance and chlorosis (sometimes called bleaching). Several genera of cyanobacteria are resistant to desiccation [2]. Under these circumstances cell structure is maintained, compatible solutes are accumulated [5] while metabolism decreases to undetectable levels. Rehydration leads to a rapid return of metabolic activity [6].

Chlorosis is the other often reported adaptation typical for vegetative cells of cyanobacteria to inadequate nutrient availability, while exposed to light. It occurs by the dismantling of the photosynthetic apparatus [7]. Cultures of several genera respond to this inadequate growth condition by acquiring a chlorotic phenotype [8], [9] and [10]. These studies show that reversion of chlorosis can be achieved once the cultures are returned to adequate culture conditions. The reversion of chlorosis has been particularly investigated in *Synechococcus* PCC 7942 cultures. In these chlorotic cultures photosynthesis was around 0.1% [11] of the photosynthesis reported for green cultures. When returned to adequate growth conditions nearly all cells of the chlorotic cultures regained their red autofluorescence and divided after 4–5 days of incubation [12].

*Microcystis* is often reported as responsible for toxic blooms [13]. It has a complex life cycle that allows it to withstand environmental variations [14]. During spring and summer the population is mainly pelagic but is able to overwinter in the benthos and survives long periods of darkness [14]. A fermentation pathway has been demonstrated [15] and the presence of genes responsible for this pathway was confirmed [16]. Cells are able to survive in the sediment for years [17] where light intensities are insufficient to maintain cell division by photosynthesis. Benthic colonies can be resuspended and recolonize the water column [18] and [19]. Efforts to understand the life cycle in the environment are being made in order to predict bloom formation.

In our previous studies we demonstrated that *Microcystis* cultures become chlorotic when maintained under nutrient limitation. The cells in these cultures seem whole and the cultures reacquire their characteristic blue green colour when returned to nutrient replete conditions [20]. It was also demonstrated that culture medium of chlorotic cultures induced chlorosis in green nutrient replete cultures. The aim of the present work was to examine several parameters of the chlorotic cultures so as to verify whether the cells in these cultures, like reported for *Synechococcus*, could be regarded as resting stages with characteristics that would allow them to survive in a growth limited state.

## Materials and Methods

### Strain and culture conditions

*Microcystis aeruginosa* PCC 7806 is maintained in our culture collection under dim light and sub cultured every 2 months into fresh liquid double strength ASM-1 medium (2 ASM-1) [21], cultured under white fluorescent lamps to increase the density of the culture and returned to the dim light until the new subculture. Before the start of the experiments the cultures maintained under dim light were transferred to Erlenmeyer flasks containing 2 ASM-1 and cultured on a rotary shaker at 90 rpm under white fluorescent light (16 h photoperiod). For the experiments these cultures, while in the growth phase, were inoculated into new 2 ASM-1 (+N) or 2 ASM-1 lacking the N source (-N, NaNO_3_) at an OD_760_ of 0.3–0.6 and samples were taken as indicated below. For the purpose of comparison with data from the literature, when necessary, the same procedures were used for *Synechococcus* PCC 7942 cultures.

### Viability of the chlorotic cultures

One and a half mL of chlorotic cultures were centrifuged for 20 s at 10000 rpm, the culture medium was removed, the pellet was resuspended in 1.5 mL of ASM-1 and the culture transferred to glass test tubes. These cultures were kept under white fluorescent light (16 h photoperiod) on shelves and their regreening observed periodically by regeneration experiments [20] or by flow cytometry monitoring red autofluorescence (FL4 photomultiplier) emission on a FACS Calibur (BD Bioscience). The experiments were carried out using four independent cultures, examined in duplicate. Again, for the purpose of comparison, the same procedure was used for chlorotic *Synechococcus* PCC 7942 cultures.

### Isolation of genomic DNA

Total genomic DNA was extracted from chlorotic *Microcystis* cultures obtained in 2 ASM-1 medium. The protocol followed was a modification of a protocol for isolating genomic DNA from gram-negative bacteria [22]. Briefly, cells from green and chlorotic cultures were harvested by centrifugation. DNA extraction was carried out in lysis buffer (1% SDS, 0.1% sarkosil, 40 mM tris-acetate pH 8.0 and 1 mM of EDTA) by mixing during 30 min at room temperature. Subsequently, 5 M NaCl was added and the extract was centrifuged at 12000 *g* for 10 min at 4°C. The supernatant was collected and extracted by gently mixing with an equal volume of chloroform for 10 min. The mixture was centrifuged for 30 min at 12000 *g* at 4°C. The aqueous phase was transferred to a new tube and the DNA precipitated with ethanol and the pellet washed twice with 70% ethanol. The pellet was dried in a speed-vac, resuspended in TE buffer plus 1 μg/mL of RNase A and incubated for 1 h at 37°C. The quantity and quality of extracted DNA was monitored using a 260:280 nm ratio absorbance. About 2 μg of DNA were loaded on wells of a 1% agarose gel and electrophoresed for 50 min at 110 V in 1X TBE buffer (90 mM of Tris-Borate and 2 mM of EDTA). DNA was visualized after ethidium bromide staining. The experiment was carried out with cultures obtained independently (two green and two chlorotic cultures obtained in 2 ASM-1 medium).

### Polymerised glucose content

Glucose was quantified after acid hydrolysis of the biomass. Cells were separated by centrifugation from the culture medium after 8 (green culture) and 40 days (chlorotic culture) of culture. Around 10^8^ cells of each time point were resuspended in 2% H_2_SO_4_ and left for 80 min at 100°C. Glucose was determined using the Bioliquid Kit (Biodiagnóstica) as described by the manufacturer.

### Metabolite profile

Cells were harvested from cultures during the growth phase (8 days, green cultures) and chlorotic phase (40 days, chlorotic culture obtained in 2 ASM-1 medium). The cells were separated from the culture medium by centrifugation, freeze dried and kept at -18°C until extraction. Freeze dried biomass (500 mg) was successively extracted with 30 mL of solvents of decreasing polarity: water, MeOH and CHCl_3_. Aqueous extracts were freeze dried and the methanolic and the chloroformic extracts were dried under reduced pressure. ^1^H NMR spectra (JEOL Eclipse + 400 MHz, operating at 400 MHz for ^1^H) were obtained after dissolving the extracts with the corresponding deuterated solvents.

### Flow cytometry experiments

All measurements were carried out on a FACS Calibur (BD Bioscience) flow cytometer. Cells were harvested from the cultures and their red autofluorescence was monitored (Fl4 photomultiplier). Nucleic acids detection in the cells was carried out by adding SYTO 9 or propidium iodide (PI) to the cultures and monitoring respectively green or red fluorescence (FL1 or FL2 photomultiplier). Concentration of the reagents was in accordance to the specifications of the supplier (Live/Dead BacLight kit from Invitrogen). To monitor esterase activity chlorotic (obtained in 2 ASM-1 medium) and green cultures were centrifuged and the supernatant was discarded. The pellet was resuspended in a 25 μg/mL solution of fluorescein diacetate (FDA) dissolved in ASM-1 (prepared by diluting a stock solution of 5 mg/ml of fluorescein diacetate in acetone). Green fluorescence (FL1 photomultiplier) of both cultures was monitored periodically by analysing 5 x 10^4^ cells on the FACS Calibur until equilibrium of the reaction was achieved. All figures represent typical results of the experiments.

### Concentration of the red autofluorescent population of chlorotic cultures and detection of chlorophyll a

The concentration of the minute autofluorescent population was carried out by density gradient centrifugation formed *in situ*. For this, 650 mL of chlorotic cultures (obtained in 2 ASM-1 medium) were concentrated by centrifugation at 3500 rpm. The supernatant was discarded and the biomass was resuspended in 0.15 M NaCl. For the density gradient centrifugation, this suspension was mixed with Percoll in NaCl 0.15 M at a proportion of 6:4. The mixture was centrifuged in an angle head rotor for 10 min at 20 000 g. The narrow band containing green cells in suspension was transferred to a new centrifuge tube. To remove the Percoll, water was added to the sample and the sample centrifuged for 30 s at 13 000 rpm. The supernatant was removed and the pellet was freeze dried.

To examine the presence of chlorophyll a, the biomass isolated was extracted in the dark with 90% MeOH. The cell debris were removed by centrifugation and the spectrum of the extract (400–710 nm) was obtained in a Shimadzu UV Mini spectrophotometer using a 10 μL cuvette with a 5 mm pathlength. This spectrum was compared to the spectrum obtained from an extract of cells from green cultures.

## Results and Discussion

### Chlorotic *Microcystis* cultures retain their re-greening potential

**Fig 1:**
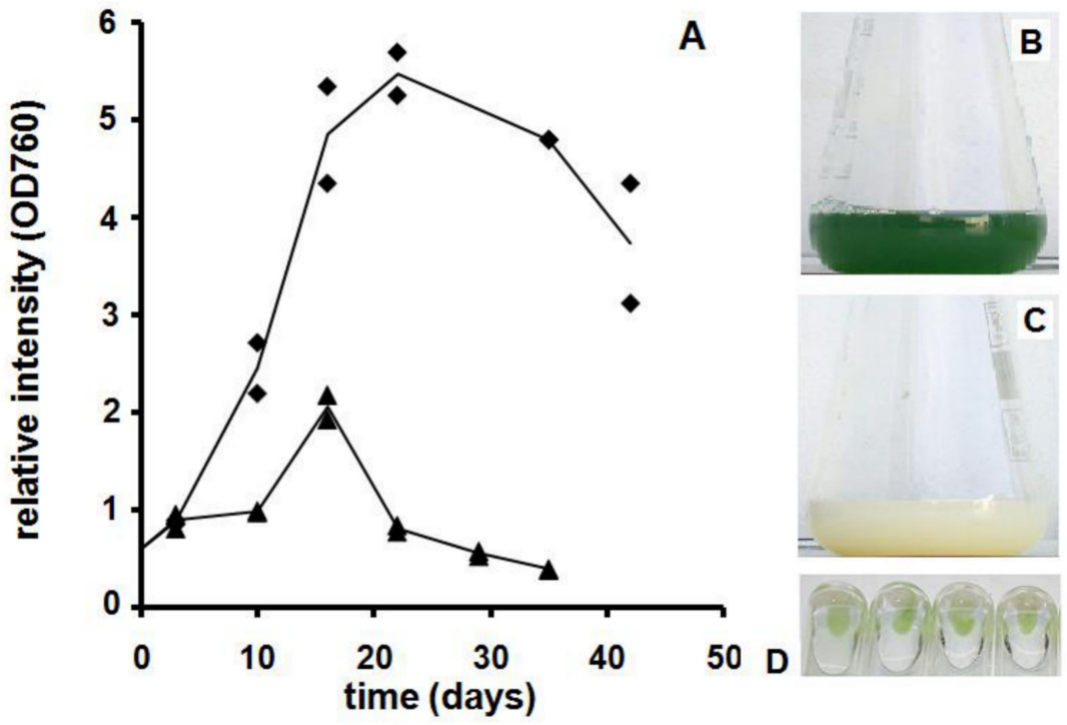
A. Growth curve of *Microcystis* PCC 7806 cultures grown in double strength ASM-1 medium with (ballons) and without N (triangles). The lines pass through the mean of two independent cultures; B. Typical colour of green culture obtained in 2 ASM-1 medium; C. Typical colour of chlorotic culture obtained in 2 ASM-1 medium; D. Regreening of chlorotic *Microcystis* PCC 7806 cultures induced by replacing the used culture medium by fresh one. Each tube contains a chlorotic culture obtained in independent flasks.

*Microcystis* PCC 7806 cultures turn chlorotic when kept in limiting nutrient conditions [20]. The timing of the bleaching of the cultures depends, among other factors, on the initial nutrient availability and on light intensity. Cultures inoculated in 2 ASM-1 medium grew during 20 days while cultures without N stopped growing after 15 days (Fig 1A). After the growth of the cultures ceased, the characteristic green colour of the cultures faded until they became completely chlorotic (S1 and S2 Figs) Fig 1B and 1C illustrate the appearance of green and chlorotic cultures, respectively. Due to the initial N limitation of the cultures inoculated in 2 ASM-1 lacking N, the cell density achieved by these cultures was of course far below that of the cultures maintained in 2 ASM-1. Nitrate, phosphate and sulphate levels were monitored in the cultures grown in 2 ASM-1 medium: when cultures reached the maximum OD_760_ phosphate and sulphate levels were around 50% of the original level while nitrate had been exhausted from the culture medium (S1 Fig).

Chlorotic cultures contain cells and the culture medium is clear with negligible amounts of cell debris. Investigation by electron microscopy [20] showed that cells from chlorotic cultures could be distinguished from the ones of green cultures clearly by their lack of thylakoid membranes. When returned to adequate growth conditions chlorotic *Microcystis* cultures, obtained both in 2 ASM-1 and 2 ASM-1 medium lacking N, regain their characteristic green colour (Fig 1D). The observations listed above are in accordance to what has been reported previously for other cyanobacterial genera submitted to nutrient starvation [7], [8] and [23].

### Chlorotic cells of *Microcystis* contain C reserves

Since chlorotic cyanobacterial cultures regain their characteristic green colour when returned to adequate conditions, chlorotic cells are considered a resting stage (or dormant like state) in their life cycle and an adaptive response to a sustained stress environment (starvation combined with light) [7]. It is expected that resting cells necessarily contain DNA and carbon reserves. Both characteristics allow cell survival in adverse conditions and a rapid repopulation in post stress environments [24].

**Fig 2:**
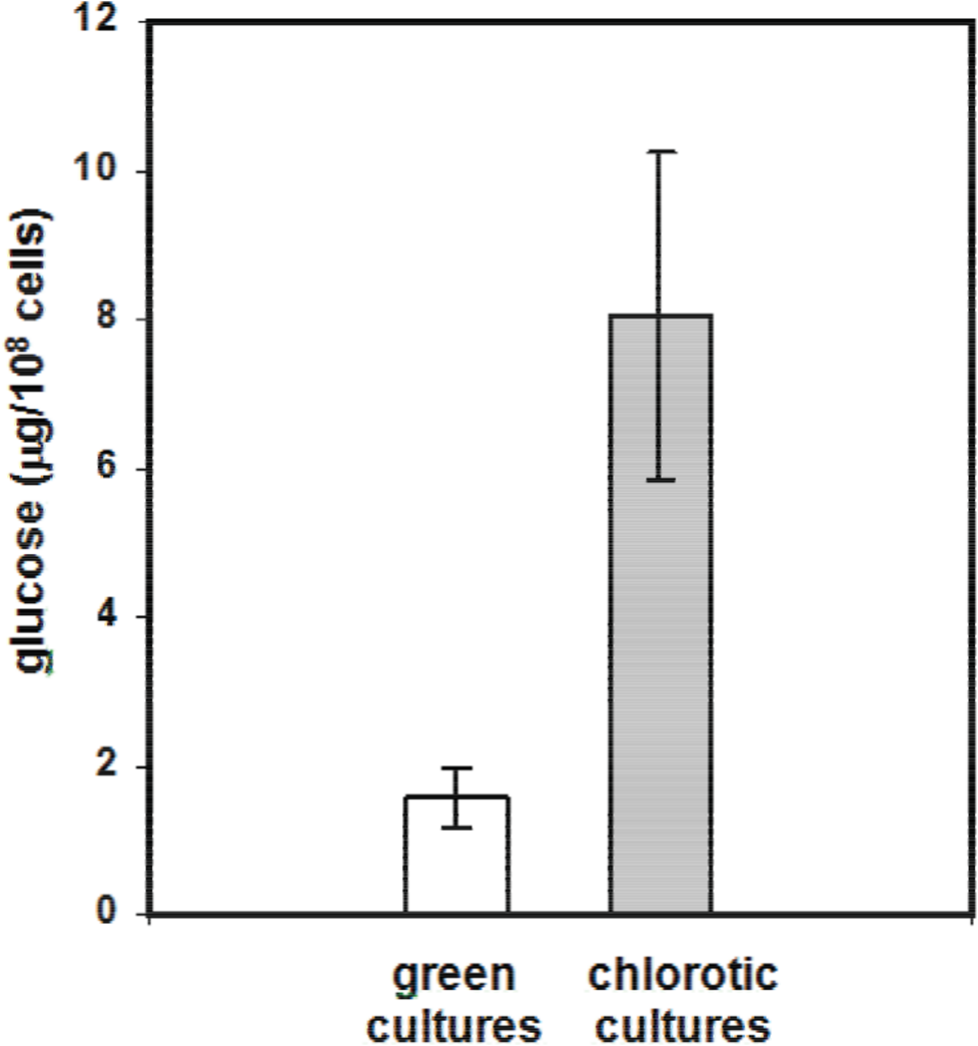
Polymerized glucose content of green (8 days of culture) and chlorotic (40 days of culture) cells from *Microcysits* cultures grown in 2 ASM-1 medium.

Chlorotic *Microcystis* cultures obtained in 2 ASM-1 medium were analysed for the presence of C reserves in the form of glucose polymers. Chlorotic cells contained around five times more polymerised glucose than green cultures in the growth phase (Fig 2). Glucose is frequently polymerised to glycogen in cyanobacteria. High levels of glycogen in chlorotic cultures have been documented in other cyanobacterial genera as a response to starvation. An example is the culture of *Synechococcus* PCC 7942 that, when deprived of N under light, becomes chlorotic and accumulates around four times more glycogen than the original green culture [12].

Poly-3-hydroxybutyrate (PHB) storage in bacteria is also a common response to starvation conditions and it has been reported to not only serve as C storage but to also alleviate a series of environmental stresses [25]. Metabolite profiling using ^1^H NMR showed a clear signal for PHB in chlorotic cultures while in green cultures this metabolite was hardly detectable (Table 1 and S3 Fig).

**Table 1:** Signal assignments and relative contents of compounds identified in the ^1^H NMR spectra of different extracts.

	label	chemical shift	green cultures*	chlorotic cultures*	compound
AQUEOUS EXTRACT	a	1.2, 2.5, 4.15	5	2	β-hydroxy butyric acid
	b	3.25	2	0	glycinebetaine
	c	1.35	0.7	0.1	lactic acid
	d	1.9	2	3	acetic acid
	e	3.5–4.0, 5.4	12	0.2	sugars
METHANOLIC EXTRACT	d	2.9	1.3	5	acetic acid
	e	3.5–4.0	4	0.5	sugars
	h	8.55	0.1	1.6	formic acid
	i	5.96, 6.16, 8.03, 8.45, 9.35, 9.6	0.8	0	chlorophyll a
	j	5.3, 5.4	9	3	unsaturated fatty acids
	k	0.9, 1.3	16	17	saturated fatty acids
CHLOROFORMIC EXTRACT	k	0.9, 1.3	1.5	1.7	saturated fatty acids
	l	1.28, 2.55, 5.26	traces	0.2	poly-β-hydroxy butyric acid

* Intensities were measured relative to the internal standards, Trimethylsilyl Propionic-2,2,3,3,-d4 acid (TMSP) and Tetramethylsilane (TMS).

^1^H NMR metabolite profiling also showed, as expected, chlorophyll a in green cultures while this compound was below the detection limit in the chlorotic ones Again, this is in accordance with results obtained for several other cyanobacterial genera as a response to nutrient starvation [7]. No other aromatic signals were detected in the spectra of the chlorotic cultures, thus no colourless chlorophyll catabolites [26] could be detected, indicating the complete breakdown of the porphyrin ring system of chlorophyll. As found for chlorophyll most of the other micromolecules identified were found in lower concentration in the cells of chlorotic cultures.

### Chlorotic *Microcysits* cultures contain two distinct populations

**Fig 3:**
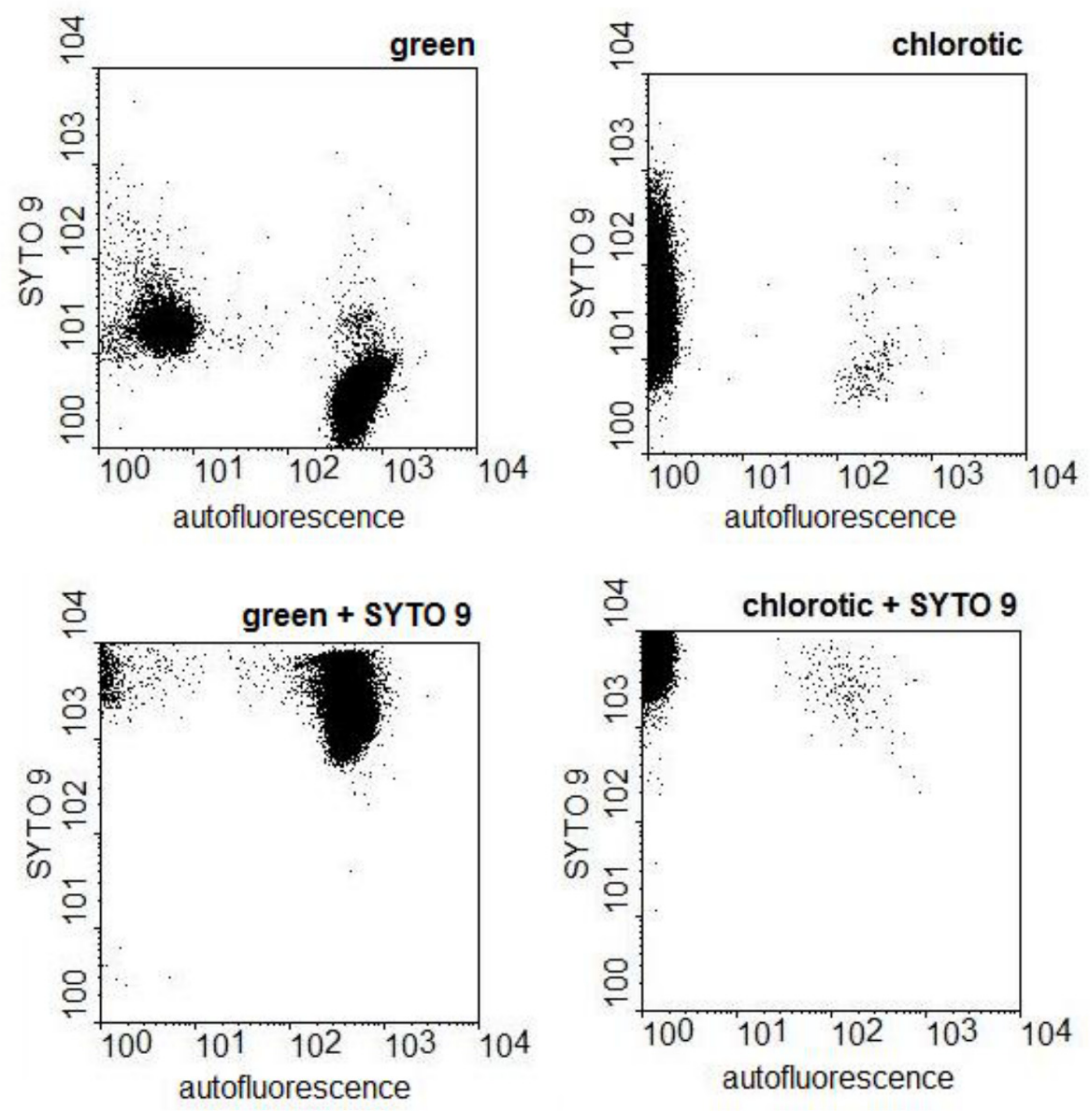
Detection of nucleic acids in *Microcystis* cells grown in 2 ASM-1 medium by flow cytometry. The dot plots show the emission strengths of the cells before and after the addition of SYTO 9 to green and to chlorotic cultures.

The nucleic acid content of the cells was analysed by flow cytometry. After adding SYTO 9, a green fluorescent nucleic acid stain permeable to cell membranes, the great majority of the cells of chlorotic cultures obtained both in culture medium with or without N emitted green fluorescence, demonstrating the presence of nucleic acids. The emission intensities of cells of chlorotic cultures were equivalent to those found in cells of green cultures (Fig 3) suggesting equivalent nucleic acid content. Equivalent results were obtained for chlorotic cultures obtained in 2 ASM-1 medium lacking N.

**Fig 4:**
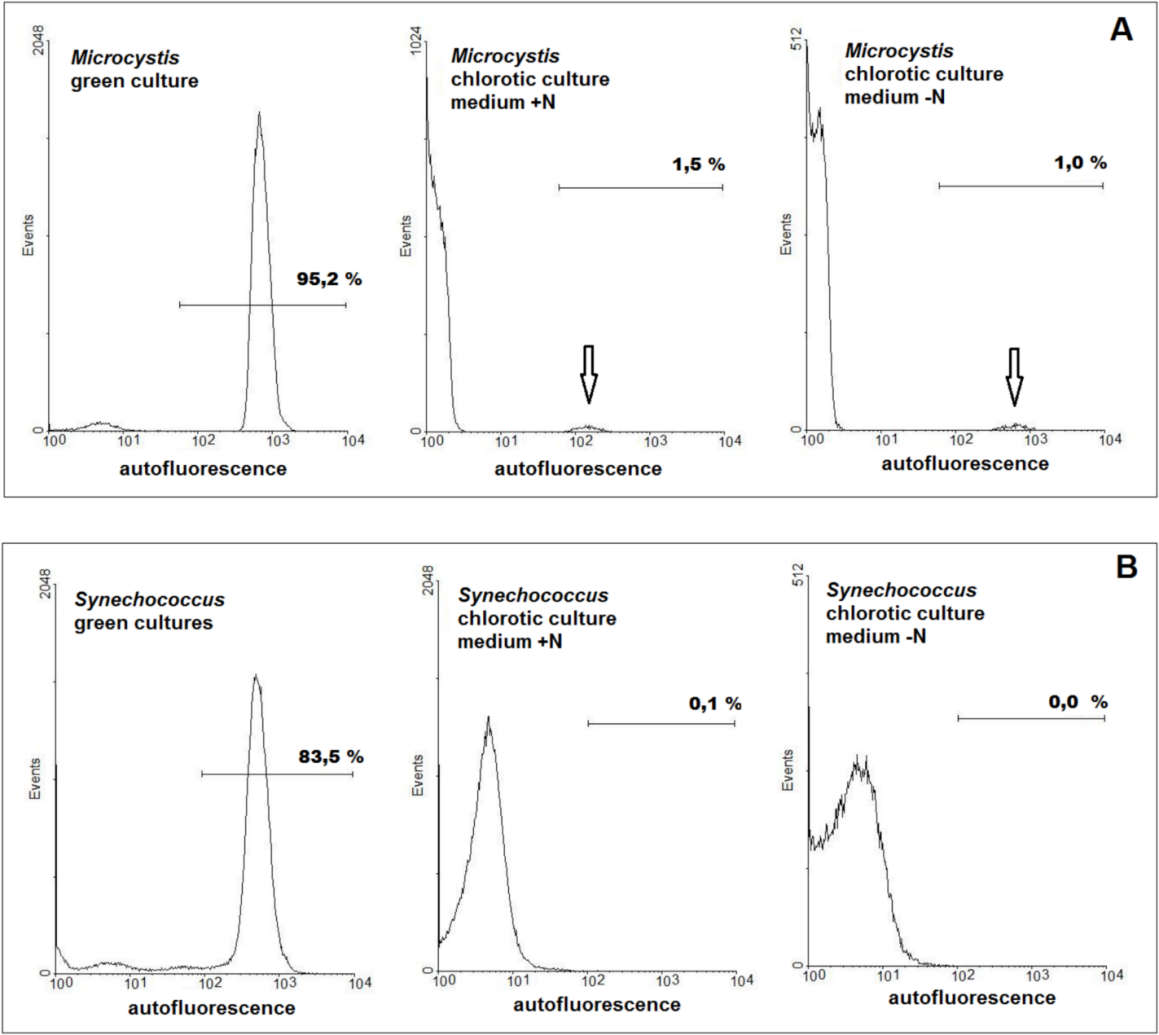
Histograms showing the red autofluorescence of the cells in green and chlorotic *Microcystis* and *Synechococcus* cultures. Cultures were grown in double strength ASM-1 medium with (+N) and without (-N) nitrogen. The arrows indicate minute populations of autofluorescent cells in chlorotic *Microcystis* cultures.

Flow cytometry also allowed single cell monitoring of red autofluorescence typical of photosynthetic cells. As expected in green cultures the great majority of the cells were red autofluorescent due to the presence of chlorophyll. In the chlorotic cultures, obtained in medium with and without N, the surprising observation was that two clearly distinct populations were present (Fig 4A). In accordance with the results obtained by ^1^H NMR the vast majority of the cells showed no red autofluorescence since no chlorophyll was detected in them. Nevertheless, a small percentage of cells in these cultures did emit red autofluorescence. Investigation of several independent chlorotic cultures obtained in similar experiments showed that the great majority of these cultures had a small red autofluorescent population of around 1% of the total population. Again the same characteristic was found in chlorotic cultures obtained in 2 ASM-1 medium lacking N: the great majority of the population had lost most of its autofluorescence while a minute population of around 1% had higher emission levels (Fig 4A). There was though a difference between the autofluorescent population of the chlorotic cultures obtained in +N and–N medium: when obtained in–N the autofluorescent population emitted at a similar level than the green cultures while the ones obtained in +N medium the autofluorescence levels were clearly lower. This difference in the autofluorescence of the cells could be clearly observed by epifluorescence microscopy: most cells in the growth phase emitted bright red fluorescence. Under the same analytical conditions in chlorotic cultures, obtained in 2 ASM-1 medium lacking N, only a few cells emitted red fluorescence at hardly detectable levels.

Red autofluorescent cells were also found in chlorotic cultures maintained for more than one and a half year under light and nutrient limited conditions, but when put under dark conditions, the number of autofluorescent cells gradually decreased.

Investigation of chlorotic *Synechococcus* cultures, obtained both in medium with and without N, showed that this genus reacted different from *Microcystis*: only a single population was found with chlorophyll emission levels lower than the found in green cultures (Fig 4B). The results obtained for *Synechococcus* are in accordance with the literature [11, 12]. In these reports it was verified that the chlorotic population had lower chlorophyll levels, was homogenous and the cells regreen and start cell division once returned to nutrient sufficient conditions.

The comparison of the results from *Microcystis* and *Synechococcus*, under the same experimental conditions, reassured that the strains from these genera do indeed react differently to nutrient limitation.

### Most of the population of chlorotic *Microcystis* cultures is dead

Since two distinct populations were observed in the chlorotic *Microcystis* cultures the question arose on how these cultures turned green again, after being added to fresh culture medium: would it be due to the reversal of the chlorotic phenotype of part of the population and/or due to the proliferation of the small autofluorescent population present initially? To better accompany the regreening of these cultures, chlorotic cultures were inoculated in to fresh culture medium and samples from these cultures were taken periodically. The autofluorescence of the cells of the culture was monitored by flow cytometry.

**Fig 5:**
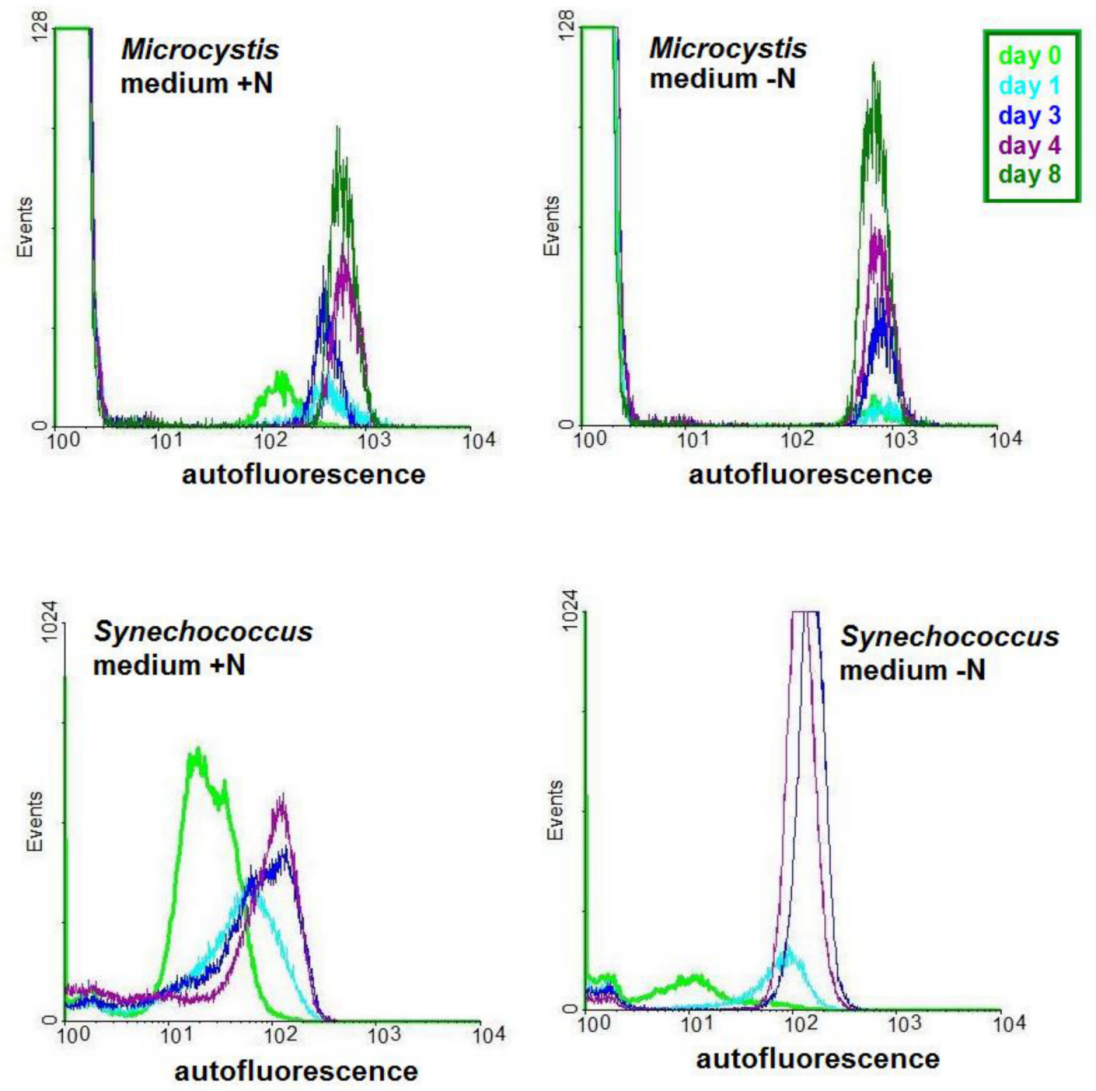
Monitoring the red autofluorescence of the cells at different time points during the regeneration experiment with chlorotic *Microcystis* and *Synechococcus* cultures by flow cytometry. The chlorotic cultures used in the regeneration experiments were obtained in double strength ASM-1 medium with (+N) or without (-N) nitrogen.

Along the experiment still two clearly distinct populations could be observed. Most of the population had red autofluorescent levels below 10 but a small population exhibited fluorescent levels above 10^2^ (Fig 5A). On the third day the number of cells of this small population had clearly increased but, until the 8^th^ day, no significant increase in autofluorescence occurred in the population with low autofluorescence, both in chlorotic cultures obtained in +N or–N medium. Nevertheless there was a small difference in the regreening of the chlorotic cultures obtained in +N and–N medium. In cultures obtained in +N first, the emission intensity of this population increased to the level of cells in green cultures and then the cell number of this population started to increase.

**Fig 6:**
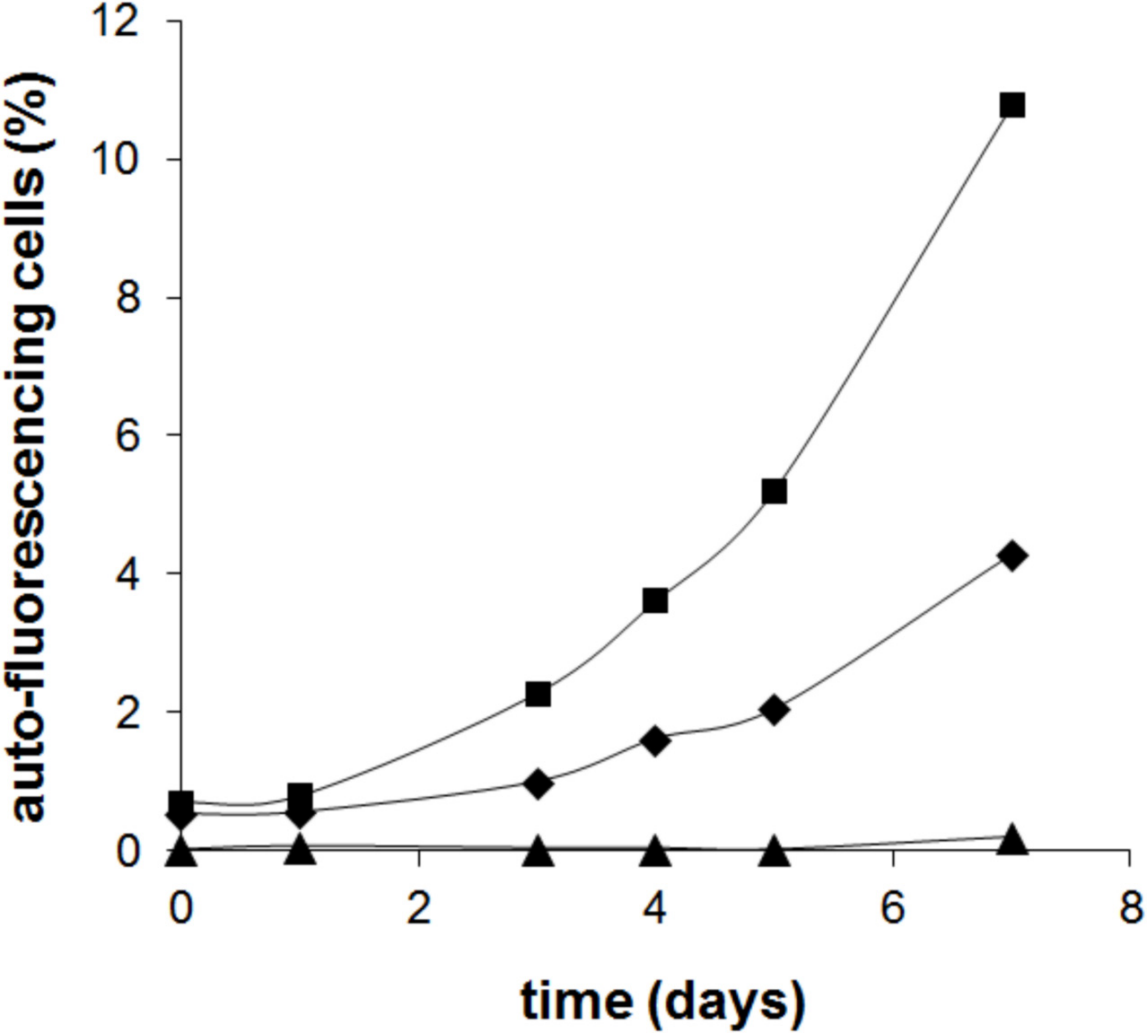
Time course of re-greening of chlorotic *Microcystis* cultures obtained in 2 ASM-1 medium. Cells from chlorotic cultures obtained in double strength ASM-1 medium were harvested by centrifugation and the spent medium replaced by fresh ASM-1 culture medium. The percentage of red autofluorescent cells in the culture was monitored by flow cytometry. Each curve corresponds to the regreening of independent chlorotic cultures.

The initial percentage of red autofluorescent cells in the population was around 1%. The number of these cells in the population increased steadily with incubation time. The time it took for the cells to begin to divide (lag phase) seemed to be dependent on the initial percentage of autofluorescent cells of the population (Fig 6). The increase in the number of red autofluorescent cells was comparable to duplication times reported for *Microcystis*, that is around 24–48 h [27]. Thus it was very probable that the increase of this population was due to the division of the original autofluorescent population already present at the beginning of the experiment.

A different situation was observed for *Synechococcus*: during the regeneration experiment there was a gradual increase in the autofluorescence of most of the population (Fig 5). This behaviour was observed in the chlorotic cultures obtained in medium with or without N, indicating that the vast majority of the population in these cultures is metabolically active and thus alive. In the cultures obtained in medium lacking N there was a clear increase in the number of cells while in the cultures obtained in medium with N no clear increase was observed. Since the experiments were carried out in a small volume of medium (1.5 mL), due to the higher densities of the *Synechococcus* chlorotic cultures obtained in 2 ASM-1 medium, the amount of nutrient was sufficient only for the reversal of chlorosis but not for the growth of the population.

From the results above we can conclude that chlorotic *Synechococcus* behaves differently from chlorotic *Microcystis* cultures. Thus in *Microcystis* it is possible that the cell division of only the small population of red autofluorescent cells initially present in the chlorotic cultures and not the reversal of chlorosis of the majority of the cells of the population is responsible for the re-greening of the culture when it is returned to nutrient rich medium.

**Fig 7:**
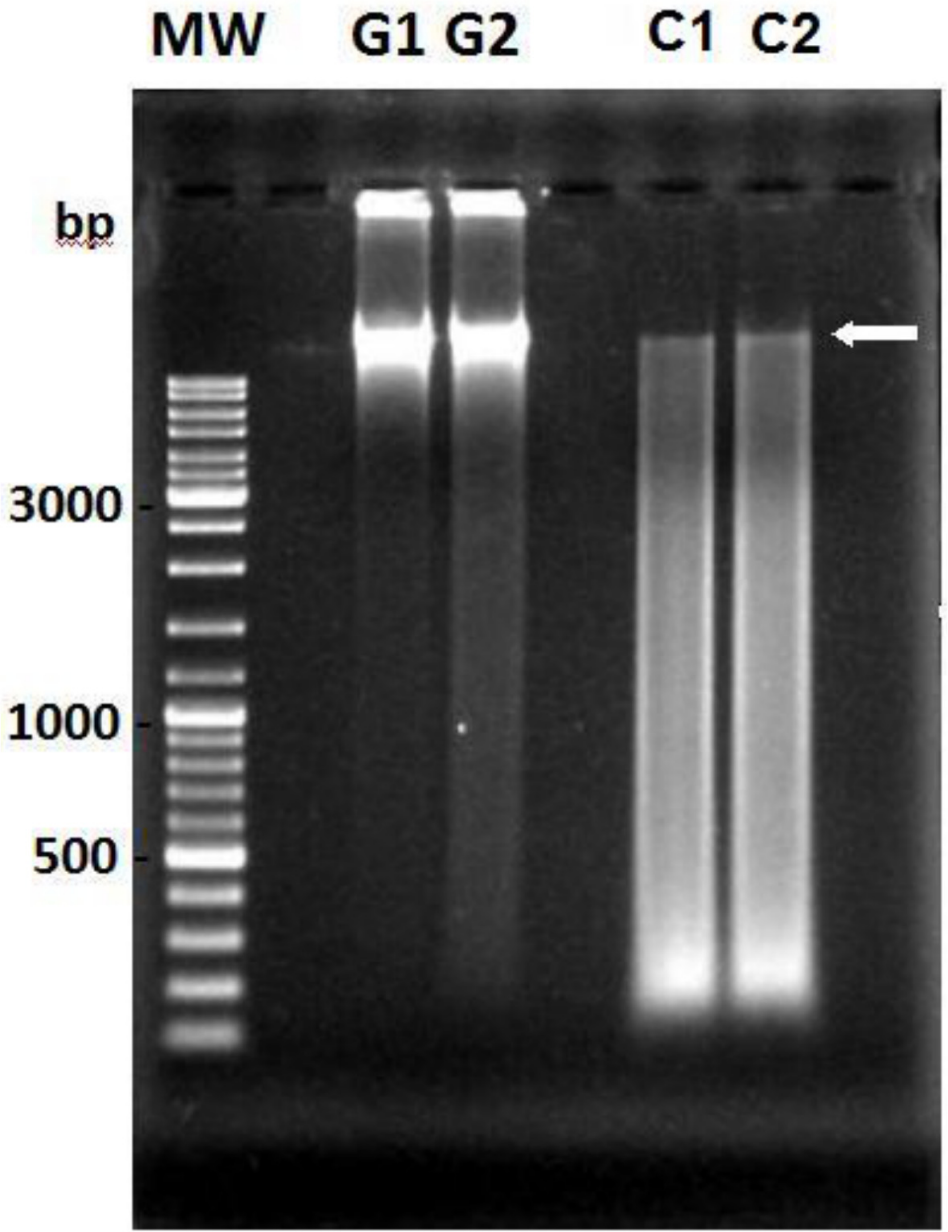
DNA integrity of cells from two independent green (G) and chlorotic (C) *Microcystis* cultures obtained in 2 ASM-1 medium. Genomic DNA was extracted and the integrity of the chromosome was examined by gel electrophoresis. The arrow indicates the position of the chromosomal DNA.

Since the chlorotic cells of *Microcystis* cultures retain some characteristics of viable cells, like C reserves (see Fig 2) and DNA (see Fig 3), it could still not be ruled out that the chlorotic cells would contribute to the re-growth of the culture, at least on a longer term. Although the presence of DNA in the cells had been detected in the entire population by flow cytometry using SYTO 9, its integrity was investigated by isolating total genomic DNA from green and chlorotic cultures. While DNA extracted from green cultures was detected as a unique band, characteristic of intact chromosomal DNA (Fig 7A), the DNA extracted from chlorotic cultures appeared as a smear and was thus mostly degraded. Only a weak band of intact chromosomal DNA could be observed in the extracts of the chlorotic cultures and thus only a minor part of population maintains its DNA integrity and thus can be viable.

**Fig 8:**
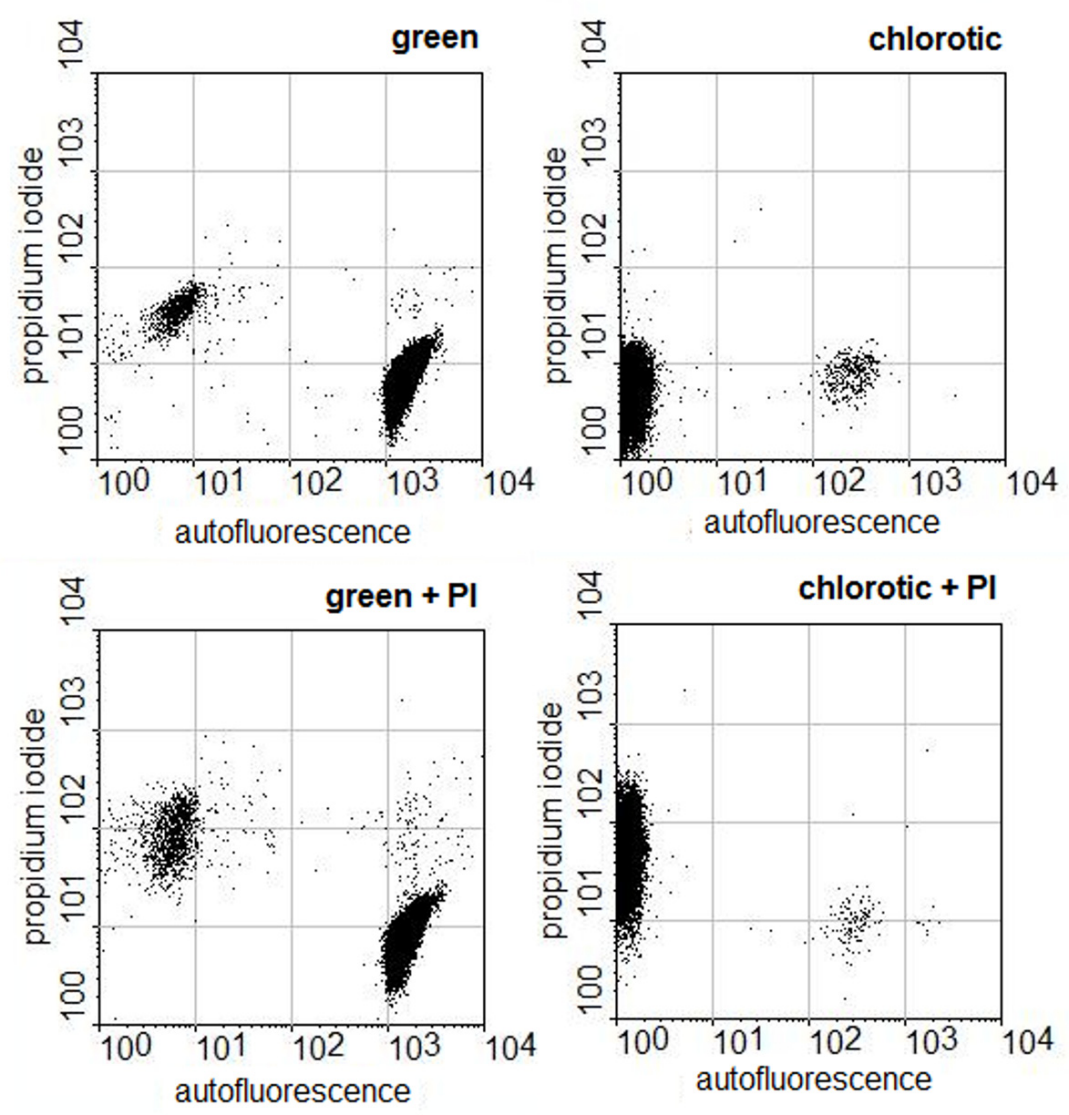
Permeability of cells of green and chlorotic *Microcystis* cultures grown in 2 ASM-1 medium to PI. The dot plots show the emission strengths of the cells before and after the addition of propidium iodide to green and to chlorotic cultures.

In a further attempt to identify the viable portion of population in the chlorotic *Microcystis* cultures membrane integrity was evaluated by adding propidium iodide (PI) to the cultures. This fluorophore penetrates only cells with damaged membranes and intercalates with the DNA therein. Fig 8 shows the analysis of the population of green and chlorotic cultures before and after the addition of PI. As expected PI did not penetrate the great majority of autofluorescent cells of both green and chlorotic cultures confirming their viability. In the subpopulation of cells of chlorotic cultures that did not autofluoresce the cell membrane was permeable to PI.

Both from the DNA and membrane integrity assessment of chloric cultures it became clear that most of its population of chlorotic *Mirocystis* cultures is not viable. The assessment of membrane permeability reassured that the autofluorescent cells are the viable portion of the population. Thus, the chlorotic cells are not resting stages in the *Microcystis* PCC 7806 life cycle and the reversion of chlorosis occurs due to the division of the small red autofluorescent population.

**Fig 9:**
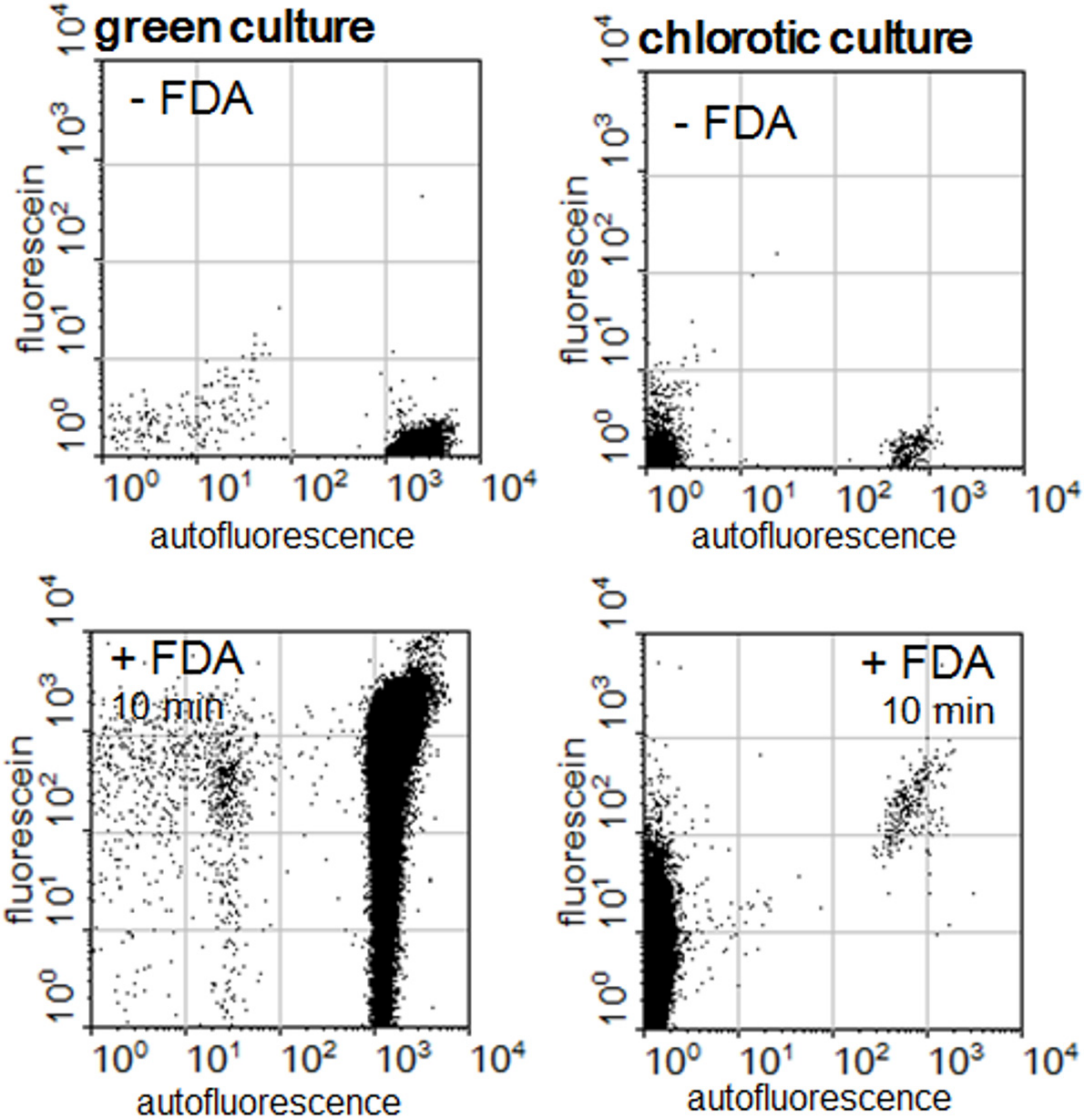
Determination of metabolic activity of green and chlorotic *Microcystis* cultures grown in 2 ASM-1 medium by the conversion of fluorescein diacetate (FDA). The dot plots show the emission strengths of the cells before and after the addition of FDA to green and to chlorotic cultures.

A comparison of the red autofluorescent cells of green and chlorotic cultures obtained in 2 ASM-1 medium showed they were different with regard to metabolic activity. First, emission intensities of red autofluorescence were lower in the cells of chlorotic cultures. Furthermore, the small population of red autofluorescent cells of the chlorotic cultures were very homogeneous with respect to fluorescein diacetate esterase activity: the great majority of this population increased their green emission with similar intensity. No such homogenous reaction levels were found in the red autofluorescent cells of green cultures (Fig 9).

### The minute population of auto-fluorescent cells contains chlorophyll a

The results above reveal clearly that the majority of the population of the chlorotic cultures is dead. Analysis by flow cytometry revealed that the minute population of red autofluorescent cells in chlorotic cultures, obtained in 2 ASM-1 medium, had lower fluorescence levels than the found in cells of green cultures. Though no chlorophyll a had been detected in the NMR spectra in the chloroform extracts of these cultures, the presence of this pigment cannot be ruled out since the concentration of red autofluorescent cells in the sample was very low (around 1%). Thus, the nature of this red autofluorescence was further investigated.

Chlorotic cultures were submitted to density gradient centrifugation using Percoll PLUS. According to the suggestion of the manufacturer, a model experiment was carried out in an attempt to separate the populations. A protocol was developed in which most part of the population sedimented (white pellet), while a thin but clear green band stayed in suspension. The pellet obtained after sedimentation and freeze drying of this band contained a clearly green pigment (S4 Fig)

The spectra of the extracts of these concentrated cells were compared to spectra obtained from biomass of green cultures. Both types of cells showed the characteristic absorption maxima of chlorophyll (437, 617 and 665 nm.). There was though a small difference between the two spectra: the spectra of the extracts from the concentrated cells showed a larger shoulder at 474 nm that corresponds to the absorption region of carotenoids.

Since chlorophyll a was detected in the cells and since the population starts to divide once it is returned to nutrient sufficient conditions it is very probable that the red autofluorescent population of chlorotic cultures contains an organized photosynthetic apparatus. Nevertheless it cannot be ruled out that the cells maintain their viability also by absorbing organic nutrients from the culture medium. As expected from a culture medium where the majority of the cells is dead, the culture medium of chlorotic *Microcystis* cultures is very complex containing a variety of organic compounds (results not shown). Facultative photo and chemo-heterotrophy has been previously reported in certain strains of the phylum [1].

### Final remarks

From the results, it can be concluded that, as found for other cyanobacterial genera, nutrient limitation in *Microcystis* cultures leads to chlorosis of the culture. Nevertheless, contrary to what has been reported for other genera, only a minute population of viable cells maintain their viability for long periods under nutrient limited conditions and the majority of the cells found in the cultures are dead. Thus, *Microcystis* PCC 7806 responds differently to nutrient limitation than for instance *Synechococcus* PCC 7942: the majority of the cells in *Synechococcus* cultures increase their autofluorescence and start to divide when returned to adequate culture conditions [12].

In many other bacteria [24], [28–31] the response of a genetically homogenous population to environmental stress is not homogenous. Frequently only a small part of the population is able to persist under stress conditions in which the majority of the population does not survive. These cells maintain viability for prolonged periods and once returned to adequate conditions they resume division and are responsible for the re-colonization of a suitable environment. *Microcystis* PCC 7806 shows a similar pattern to nutrient stress response: the small viable population contains photosynthetic pigments and resumes cell division when returned to nutrient replete conditions.

There is an increasing need to evaluate metabolic heterogeneity of phytoplankton species in order to understand population dynamics [32]. *Microcystis* is frequently responsible for bloom formation in fresh waters and these are often toxic leading to major health [13] and environmental issues [33]. Nutrient limitation is a common stress that populations have to face in the environment. The study of *Microcystis* metabolic plasticity will bring insights to the understanding of the adaptive mechanisms that allows it to withstand unfavourable growth conditions. Metabolic differentiation in *Microcystis* will continue to be subject of our studies in order to understand the life cycle of these organisms and predict bloom formation.

## Supporting Information

S1 Fig A, Growth curve of *Microcystis* PCC 7806 culture grown in double strength 2ASM-1 medium. The points in the graph represent the growth in two independent flasks and the line passes through the mean of the measurements. B, Nutrient availability in the culture medium during culture (100% represents the concentration of the medium at the beginning of the experiment (squares NO_3_, triangles PO_4_ and balloons SO_4_.
(TIF)

S2 Fig *Microcystis* PCC 7806 cultures grown in double strength ASM-1 medium at different stages of chlorosis. (TIF)

S3 Fig Metabolic profile of green (8 days of culture) and chlorotic cultures (40 days of culture). Cells were extracted in sequence with solvents of decreasing polarity. The compounds identified and their relative contents are listed in Table 1.
(TIF)

S4 Fig Thin green pellets obtained from chlorotic cultures by density gradient centrifugation. (TIF)
