# Supplementary figures and images for: Persistence of Only a Minute Viable Population in Chlorotic Microcystis aeruginosa PCC 7806 Cultures Obtained by Nutrient Limitation
Source: PLoS One. 2015 Jul 16;10(7):e0133075. doi: 10.1371/journal.pone.0133075 (PMC4504671; doi:10.1371/journal.pone.0133075)

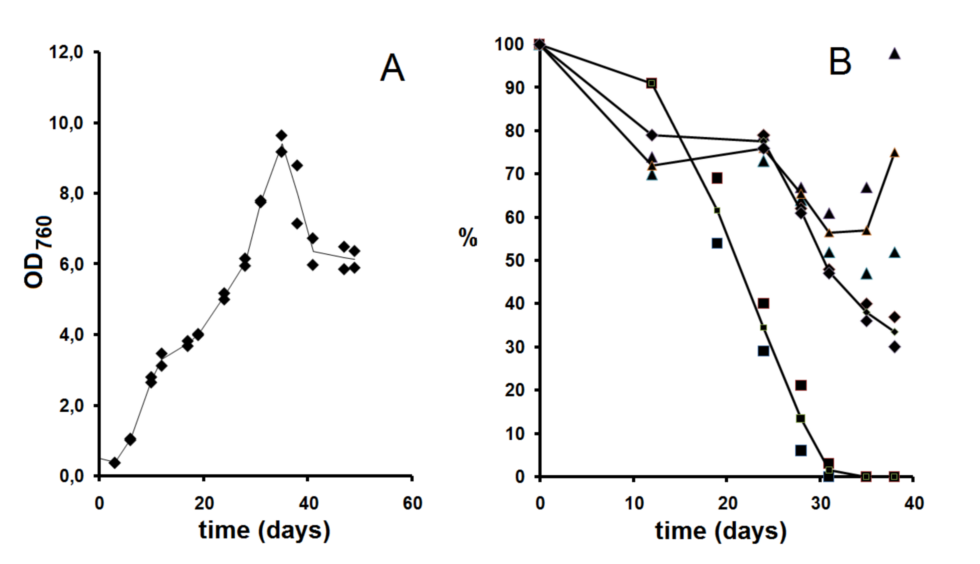

Supplement: S1 Fig — The points in the graph represent the growth in two independent flasks and the line passes through the mean of the measurements. B, Nutrient availability in the culture medium during culture (100% represents the concentration of the medium at the beginning of the experiment (squares NO3, triangles PO4 and balloons SO4. (TIF) [file pone.0133075.s001.tif]

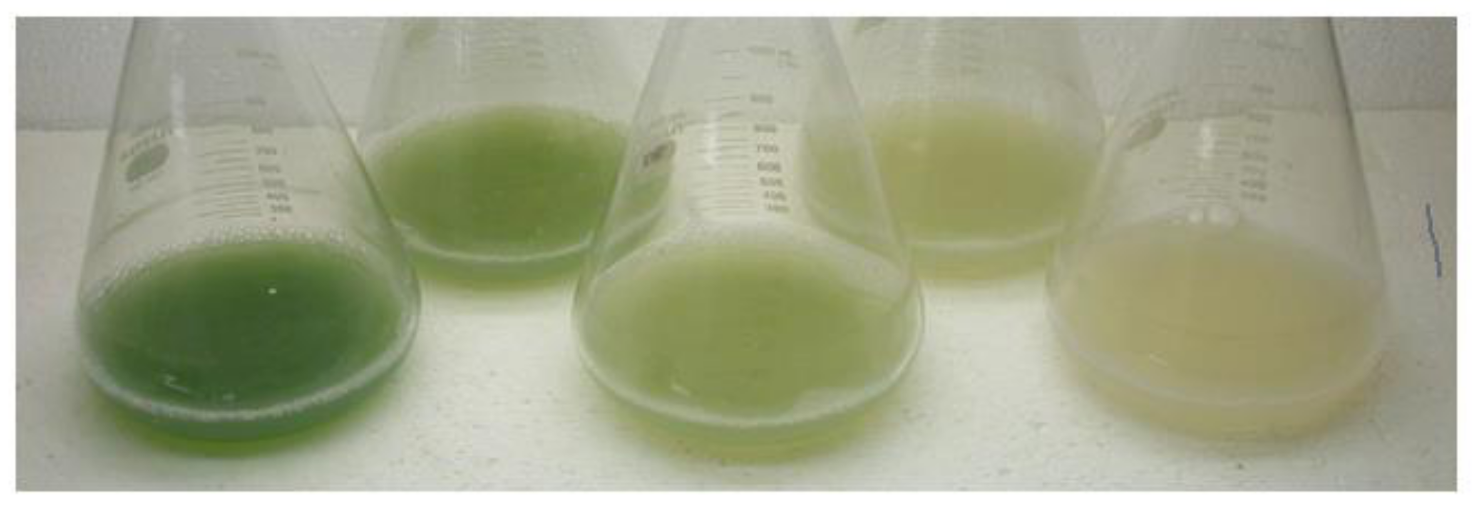

Supplement: S2 Fig — (TIF) [file pone.0133075.s002.tif]

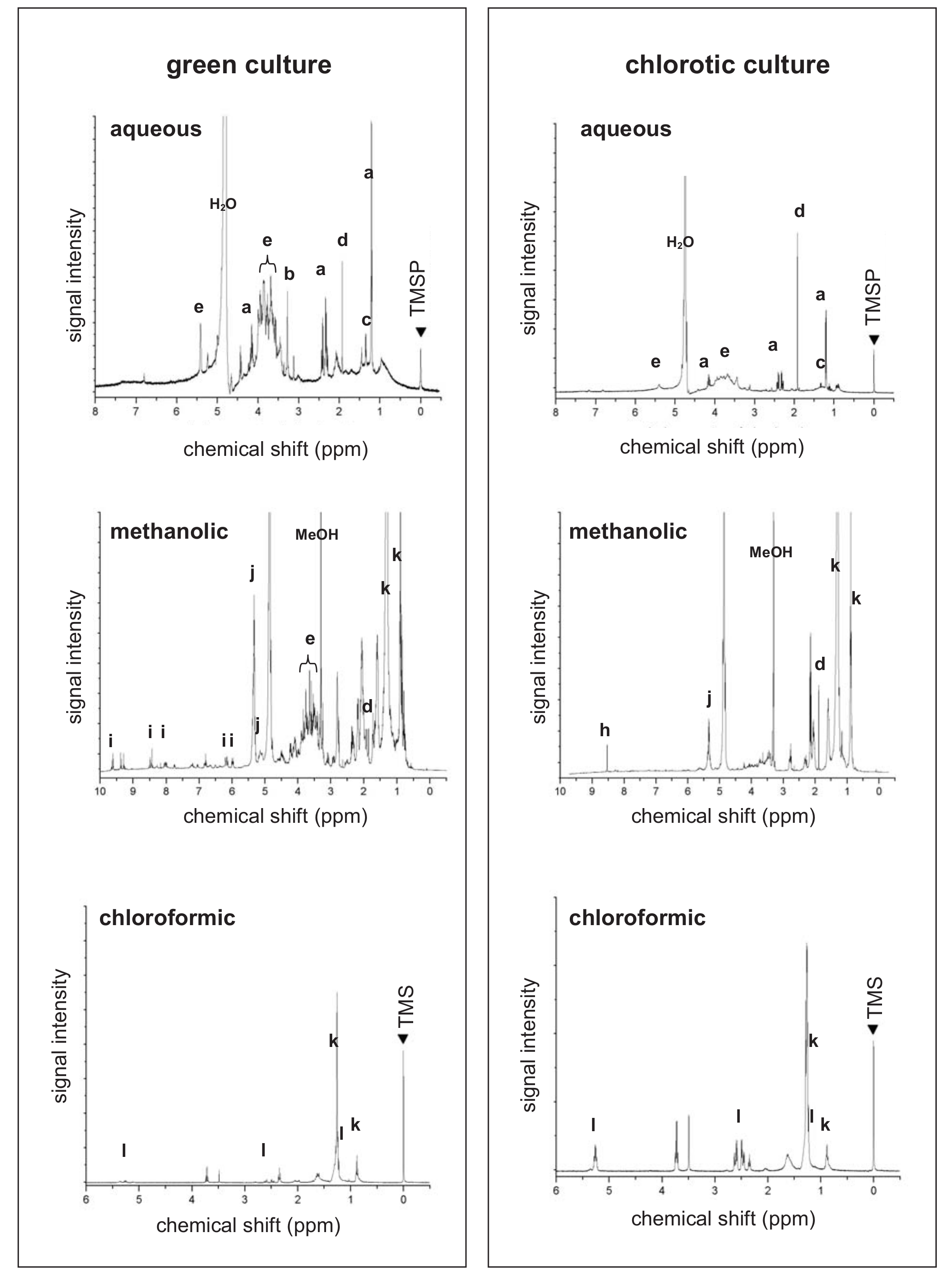

Supplement: S3 Fig — Cells were extracted in sequence with solvents of decreasing polarity. The compounds identified and their relative contents are listed in Table 1. (TIF) [file pone.0133075.s003.tif]

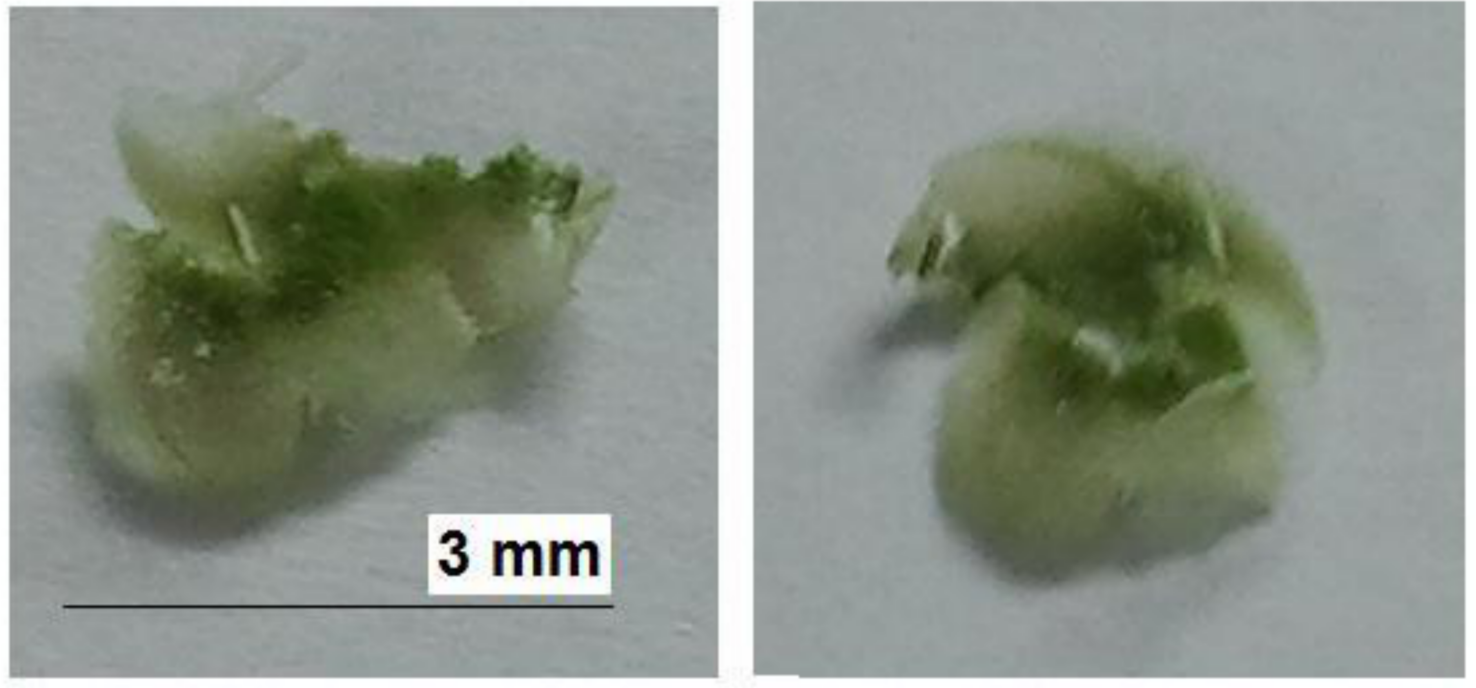

Supplement: S4 Fig — (TIF) [file pone.0133075.s004.tif]
